# Human papillomavirus (HPV) detection in vaginal self-samples: evaluation of eNat® as an alternative suspension medium to ThinPrep®PreservCyt® for vaginal swabs

**DOI:** 10.12688/openreseurope.14344.1

**Published:** 2022-03-10

**Authors:** Chiara Giubbi, Marianna Martinelli, Ivan Vallini, Silvia Paganoni, Tarig Dafa'alla, Federica Perdoni, Rosario Musumeci, Winnie Wu, Santina Castriciano, Paolo Romano, Clementina E. Cocuzza

**Affiliations:** 1 Department of Medicine and Surgery, University of Milano-Bicocca, Monza, Italy; 2 Hiantis S.r.l., Milano, Italy; 3 GeneFirst Ltd., Abingdon, OX14 3DB, UK; 4 Copan Italia SpA, Brescia, Italy

**Keywords:** Human Papillomavirus (HPV), HPV-testing, vaginal self-collection, ThinPrep®PreservCyt, eNat®

## Abstract

Human Papillomavirus (HPV) testing on self-collected samples allows for improved coverage rates of cervical cancer (CC) screening programs. ThinPrep®PreservCyt® (HOLOGIC®, USA) medium is widely used for the suspension of cervical and vaginal self-samples. However, this medium is costly, toxic, and flammable, involving special handling procedures which make its use difficult in screening programs, particularly in low- and middle-income countries.

This pilot study aimed to evaluate the analytical performance of eNat **®** (Copan SpA), an alternative non-alcohol-based suspension medium, compared to ThinPrep®PreservCyt® (HOLOGIC®) for high-risk HPV (hrHPV) detection in vaginal self-collected swabs using three different real-time polymerase chain reaction (RT-PCR) HPV assays: Anyplex™II HPV28 (Seegene, Korea), Papilloplex® High Risk HPV (GeneFirst, UK), and HPV OncoPredict (Hiantis, Italy).

30 women, referred to colposcopy, were enrolled in this observational, prospective pilot study and asked to collect two vaginal self-taken samples, which were suspended in 5 mL of ThinPrep®PreservCyt® or eNat®. Nucleic acids were extracted from 200 μL using Microlab Nimbus platform (Seegene, Korea) and tested with the three different RT-PCR full-genotyping high-risk HPV assays. The HPV results of vaginal samples resuspended in the two different media were compared to those obtained from the reference clinician-collected cervical sample from the same woman.

hrHPV detection in vaginal self-samples suspended in both media demonstrated a substantial agreement with cervical samples with the three assays under-investigation (0.667 <k <0.796). Moreover, the discordances between vaginal self-samples collected from the same woman were found only in cases of normal cytology or low-grade cytological lesions and were generally related to low hrHPV viral loads as indicated by the quantitative HPV OncoPredict assay (6.24E+02 copies/10,000 cells).

This study demonstrated a very good agreement between cervical and vaginal self-collected samples suspended in ThinPrep®PreservCyt® and eNat®, suggesting that the latter could represent a good alternative medium in HPV screening programs based on self-collection.

## Plain language summary

Persistent long-term infection with Human Papillomaviruses (HPVs) is associated with the development of cervical carcinoma. Cervical cancer (CC) screening programs based on the detection of HPV can reduce its incidence and mortality. Screening programs, based on HPV testing of self-collected vaginal samples, have been shown to be more acceptable to women and can improve their participation to CC screening programs. ThinPrep ^®^PreservCyt ^®^ (HOLOGIC ^®^, USA) solution is widely used for the suspension of vaginal self-samples. However, this solution is costly, toxic, and flammable, requiring special handling procedures, making its use difficult, particularly in low- and middle-income countries. This study aims to evaluate an alternative non-alcohol-based suspension medium, the eNat ^®^ (Copan SpA, Italy) for HPV detection in vaginal self-collected samples. This study involved the enrolment of women referred to a gynaecologist for an abnormal Pap test. During their gynaecological visit, women were asked to provide two self-collected vaginal swabs, one to be suspended in the eNat ^®^ medium and the other in ThinPrep ^®^PreservCyt ^®^ solution, prior to HPV testing. An additional sample, collected by the clinician from the cervix of the same patients, was used as gold-standard reference method to compare HPV results obtained from the two vaginal samples. All samples were tested using three different HPV assays: a commercial kit, and two other HPV tests recently developed as part of a project financed by the European Commission. Results obtained using all three evaluated HPV tests on vaginal samples suspended in the alternative eNat ^®^ medium were comparable to those obtained on vaginal and cervical samples suspended in ThinPrep ^®^PreservCyt ^®^ solution. In conclusion, an improved and cost-effective solution for CC screening based on self-collected vaginal samples, suspended in an alternative non-flammable medium, compatible with three different innovative HPV assays, has been validated with the aim to favour women’s participation, particularly in low- and middle-income countries.

## Introduction

According to data from GLOBOCAN 2020, a total of 604,127 new cases of cervical cancer (CC) and 341,831 CC related deaths occurred worldwide in 2020. The recent call of action proposed by the World Health Organization (WHO) to eliminate CC sets as one of the goals the screening of 70% women by the age of 35 and again by the age of 45 using high-performance assays, such as human papillomavirus (HPV) testing by 2030 ^1^.

The introduction of HPV testing as an analysis tool offers the possibility of using self-sampling to increase participation in screening programs for CC prevention. HPV testing on self-samples has been reported to be similarly accurate in the detection of cervical precancerous lesions, as well as HPV testing performed on clinician-collected cervical samples. Moreover, self-sampling allows women who, for socio-cultural reasons, do not access gynaecological examination, to take part to screening programs ^2– 4^.

ThinPrep ^®^PreservCyt ^®^ (HOLOGIC ^®^, USA) is an alcohol-based solution that serves as transport and liquid preservative for performing liquid-based Pap Smear on cervical samples. Therefore, HPV testing is routinely performed on the same type of sample and, for analogy, vaginal swabs for self-sampling have been usually suspended in ThinPrep ^®^PreservCyt ^®^solution.

Because of its high percentage of methanol, ThinPrep ^®^PreservCyt ^®^ is flammable and requires special handling and additional costs for transport. These characteristics of ThinPrep ^®^PreservCyt ^®^ make it difficult to introduce its use in self-collection-based CC screening in low to middle income countries. To overcome these problems, a non-alcohol-based medium to suspend self-collected vaginal samples that supports HPV nucleic acid stability and that is suitable for molecular HPV analysis is necessary ^5^.

eNat ^®^ (Copan Italia SpA, Brescia Italy) is a lysing based molecular collection medium already used for nucleic acids amplification assays ^6– 8^. Moreover, it preserves and stabilizes nucleic acids and desaturates proteins and inactivates microbial agents potentially contained in clinical samples.

This study aims to evaluate the analytical concordance of HPV testing conducted on a physician-collected cervical sample (gold-standard) as compared to that performed on two dry vaginal self-collected samples eluted respectively in ThinPrep ^®^PreservCyt ^®^ and eNat ^®^.

## Methods

### Ethics and consent

This study was approved by The Ethics Committee of the University of Milano-Bicocca, Monza, Italy (Protocol n. 0037320/2017 and 0086409/2018). All participants gave written informed consent prior to participation.

### Study design and samples collection

30 women, referred to colposcopy for a recent abnormal Pap smear reported as either low-grade intra-epithelial lesion (LSIL), high-grade intra-epithelial lesions (HSIL), atypical squamous cells of undetermined significance (ASCUS), or atypical glandular cells of undetermined significance (ACGUS), such as ASCH (atypical squamous cells – cannot exclude HSIL) and ACG (atypical glandular cells), were enrolled as part of an ongoing study (from March 2020 to January 2021). Immunocompromised patients, women with autoimmune diseases or any diseases involving the immune system, including HIV infection, with a presumed or confirmed pregnancy, with a diagnosis of any malignancies, or undergoing or having finished a course of chemotherapy during the six months preceding the study were excluded from the study. After signing the written informed consent, women were provided with the vaginal collection devices as well as written instructions illustrating how to perform the self-sampling; medical and nursing staff were also available if further assistance was required by the participating women. All enrolled women autonomously collected two vaginal-self samples using FLOQSwab ^®^ 552C.80 device (Copan Italia SpA, Brescia Italy) prior to coloposcopic examination. The two vaginal self-collected samples were numbered to trace the order in which they were collected and kept dry at room temperature until analysis. During the colposcopy examination, a cervical sample was taken by the gynaecologist using an L-shaped Endo/Esocervical FLOQSwab ^®^ (Copan Italia SpA, Brescia Italy). Women underwent biopsy and/or conization depending on the colposcopy outcome, according to the local clinical protocol. All specimens were transported to the Laboratory of Clinical Microbiology of the Department of Medicine and Surgery, University of Milano-Bicocca, Italy, where they were processed.

### Pre-analytic sample processing

Clinician-collected cervical samples, obtained using the L-shaped swab, were immediately placed in 20 ml of ThinPrep ^®^PreservCyt ^®^. In the laboratory all specimens were vortexed for 30 seconds, and subsequently 1.5 ml aliquots were dispensed in cryovials; one was used for DNA extraction and the others stored at -20°C.

Vaginal self-collected swabs were transported dry to the laboratory. One swab was suspended in 5 ml of ThinPrep ^®^PreservCyt ^®^ and the other in 5 ml of eNat ^®^. In order to avoid bias associated with the order of vaginal swab collection, the first vaginal swab collected from 15 women was suspended in ThinPrep ^®^PreservCyt ^®^, while the second in eNat ^®^; for the remaining 15 patients the first specimen was suspended in eNat ^®^ and the second in ThinPrep ^®^PreservCyt ^®^. Five aliquots of 1 ml were made from each of the vaginal specimens; one was used for nucleic acids extraction and the others stored at -20°C.

### Nucleic acids extraction and HPV detection

One aliquot of the cervical and of each of the vaginal samples was extracted using STARMag 96x4 Universal Cartridge Kit (Seegene, Korea) on Microlab Nimbus (Seegene, Korea) platform, a completely automated Liquid Handling Workstation for nucleic acid extraction and polymerase chain reaction (PCR) setup of up to 72 specimens. DNA was extracted from a 200 μL volume of each sample and following extraction eluted in 100 μL of the kit elution buffer, according to the manufacturer’s instructions.

Cervical and vaginal specimens were tested for HPV genotypes using three different real-time PCR full-genotyping HPV assays: Anyplex™II HPV28 (Seegene, Korea), Papilloplex ^®^ High Risk HPV (GeneFirst, UK) and HPV OncoPredict (Hiantis, Italy).

The first assay can identify 14 hrHPV (16, 18, 31, 33, 35, 39, 45, 51, 52, 56, 58, 59, 66 and 68) and 14 Low-risk HPV (lrHPV) types (6, 11, 26, 40, 42, 43, 44, 53, 54, 61, 69, 70, 73, and 82) in two different reaction mixes by means of real-time PCR assays. Papilloplex ^®^ High Risk HPV is able to detect and types 14 hrHPV (16, 18, 31, 33, 35, 39, 45, 51, 52, 56, 58, 59, 66 and 68) based on specific melting profiles. HPV OncoPredict assay detects and quantifies 12 hrHPV (16, 18, 31, 33, 35, 39, 45, 51, 52, 56, 58, 59) and uses C-C chemokine receptor type 5 (CCR5) to detect sample’s cellularity both to evaluate sample adequacy and to allow for normalization of viral load.

All three Real-time assays were performed using a CFX96 PCR Thermal Cycler (Bio-Rad, Hercules, USA) according to manufacturers’ instructions using 5 μl of template DNA in a total volume of 20 μl for Anyplex™II HPV28. Analysis with Papilloplex ^®^ High Risk HPV and HPV OncoPredict was performed using 5 μl of extracted DNA in a total volume of 20 μl.

### Statistical analysis

Patients’ age was described by median value and interquartile range (IQ, range: IQ1-IQ3). Viral load was expressed as number of viral genome copies (cp)/10,000 cells. Agreement between HPV testing results on different types of samples and different tests was evaluated with the Cohen's kappa (κ) statistics using GraphPad QuickCalcs 2014 software. Agreement was defined as slight (0.00<k<0.20), fair (0.20<k<0.40), moderate (0.41<k<0.60), substantial (0.61<k<0.80) and almost perfect (0.81<k<1.00) as previously reported ^9^.

## Results

### Population analysis

The median age of the 30 women enrolled for this study was 36.5 years (interquartile range (IQ): 29.3–47). Most of the women (23/30; 76.7%) presented cytological alterations: low-grade intra-epithelial lesion (LSIL) was the most frequently detected (14/30; 46.7%) followed by the atypical squamous cells of undetermined significance (ASCUS) in 5/30 (16.7%) women. High-grade intra-epithelial lesions (HSIL) were found in 3/30 (10.0%) women, atypical glandular cells (AGC) in 1/30 (3.0%) woman and 7/30 (23.0%) women had a negative Pap smear result.

Following colposcopy examination results, 5/30 (16.7%) women underwent conization: histological result was cervical intra-epithelial neoplasia grade 1 (CIN1) for one woman, cervical intra-epithelial neoplasia grade 2 (CIN2) for two women and cervical intra-epithelial neoplasia grade 3 (CIN3) for two women.

### Prevalence of hrHPV in cervical and vaginal samples

17 out of 30 (17/30, 56.6%) cervical samples were found to be hrHPV positive using Anyplex™ HPV28 detection kit, 16/30 (53.3%) with HPV OncoPredict and 15/30 (50%) with Papilloplex ^®^ High Risk HPV. Among vaginal self-samples suspended in ThinPrep ^®^PreservCyt ^®^, 20/30 (66.6%), 19/30 (63.3%) and 20/30 (66.6%) were hrHPV positive with each of the three assays, respectively; while among those suspended in eNat ^®^ 20/30 (66.6%) were found to be hrHPV positive with all three kits.

**Figure 1.**
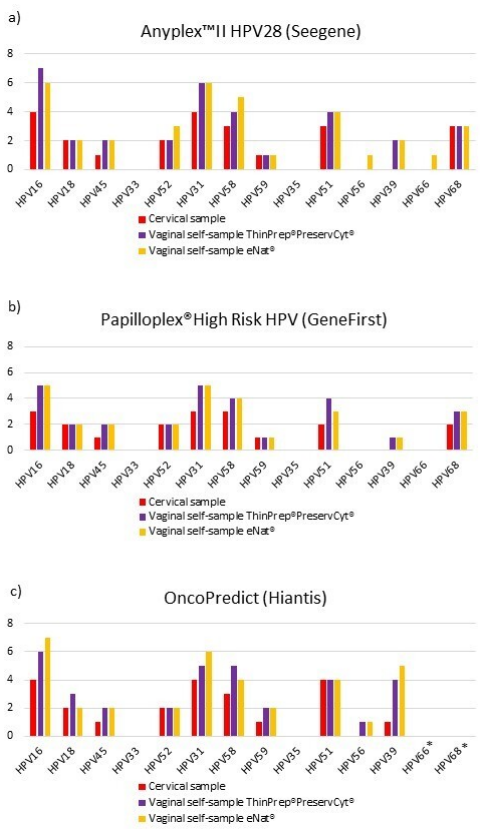
**a**) Prevalence of high-risk human papillomavirus (hrHPV) infections with Anyplex™II HPV28 (Seegene); **b**) Prevalence of hrHPV infections with Papilloplex ^®^ High Risk HPV (GeneFirst); **c**) Prevalence of hrHPV infections with HPV OncoPredict (Hiantis). *HPV Oncopredict assay does not detect HPV 66 and HPV68 genotypes.

Figure 1 shows the distribution of different hrHPV genotypes among cervical and vaginal samples according to the three different HPV assays. HPV16 and HPV31 were the hrHPV types most frequently detected with the three different methods, in cervical and vaginal-self samples suspended in ThinPrep ^®^PreservCyt ^®^ and eNat ^®^. None of the enrolled women were found to be positive for HPV33 or HPV35.

### hrHPV detection agreement

As reported in Table 1, for all HPV DNA kits, a substantial agreement for the detection of any hrHPV between vaginal swabs eluted in either eNat ^®^ or ThinPrep ^®^PreservCyt ^®^ media and cervical samples was observed.

**Table 1.** Agreement between clinician collected cervical samples and vaginal self-samples suspended in ThinPrep ^®^PreservCyt ^®^ and eNat ^®^.

	Anyplex™II HPV28 (Seegene)		Papilloplex® High Risk HPV (GeneFirst)		HPV OncoPredict (Hiantis)	
	Vaginal self-samples ThinPrep ^®^PreservCyt ^®^	Vaginal self-samples eNat ^®^	Vaginal self-samples ThinPrep ^®^PreservCyt ^®^	Vaginal self-samples eNat ^®^	Vaginal self-samples ThinPrep ^®^PreservCyt ^®^	Vaginal self-samples eNat ^®^
	% concordance; Cohen k	% concordance; Cohen k	% concordance; Cohen k	% concordance; Cohen k	% concordance; Cohen k	% concordance; Cohen k
Clinician- collected cervical samples	90.0%; k=0.791	90.0%; k=0.791	83.3%; k=0.667	83.3%; k=0.667	90.0%; k=0.796	86.7%; k= 0.727

The concordance between vaginal self-samples suspended in ThinPrep ^®^PreservCyt ^®^ and eNat ^®^ was demonstrated to be almost perfect with percentages of agreement of 100.0% (30/30; k=1.000) 100.0% (30/30; k=1.000) and 96.7% (29/30; k=0.927) respectively with Anyplex™II HPV28, Papilloplex ^®^ High Risk HPV and OncoPredict.

For all HPV DNA tests, no differences in HPV detection rate related to the order of vaginal specimens’ collection were observed.

### Viral load quantification

HPV OncoPredict quantification kit was used to determine HPV type specific and total viral load for all tested samples.

The mean value of the total normalized viral load in cervical samples was lower than that detected in vaginal samples suspended either in ThinPrep ^®^PreservCyt ^®^ or eNat ^®^ (2.03E+05 cp/10,000 cells vs 3.26E+05 cp/10,000 cells and 4.59E+05 cp/10,000 cells; respectively). Interestingly, similar viral loads were detected in both vaginal samples irrespective of the suspension medium used. Discordant results in type-specific HPV infection between the two vaginal swabs/woman were associated with either low-grade or negative cytology or with viral loads that were below 6.24E+02 cp/10,000 cells.

## Discussion

This study compared the analytical concordance of hrHPV DNA detection in self-collected vaginal swabs resuspended in two different media (eNat ^®^ and ThinPrep ^®^PreservCyt ^®^) as compared to that detected in clinician-collected cervical samples.

Tested cervical specimens were found to be hrHPV positive with a percentage ranging from 50% to 56.6% using the three investigated hrHPV detection kits; whilst a range of 63.3% to 66.6% positivity was observed among vaginal swabs. HPV16 and HPV31 were shown to be the most prevalent hrHPV types in both cervical and vaginal samples, as also previously reported ^10, 11^. The slight variation in the distribution of hrHPV types may be due to the difference in the anatomical sites of samples’ collection ^12, 13^, but also to the difference in the total volume used for sample suspension; cervical samples and vaginal specimens were suspended in 20 mL and 5 mL, respectively. The reduction in volume of collection medium may, in fact, improve HPV detection and, at the same time, reduce the costs of HPV screening ^5^.

In this study, substantial agreement in hrHPV detection was demonstrated between cervical and vaginal self-samples with a concordance rate ranging from 83.3% to 90.0% for the different assays and suspension media; this is in agreement with previous reports ^13– 16^ confirming that self-sampling could be a procedure to improve screening coverage rates. With growing evidence to support this alternative method for sample collection ^2, 14^, the introduction of self-sampling, as a strategy to prevent CC may increase participation of women not attending organized prevention programs, and may also be a useful alternative to perform screening in low to middle income countries where CC is still widespread [ GLOBOCAN, 2020]. However, the higher costs associated with the use of ThinPrep ^®^PreservCyt ^®^ together with its flammable nature may delay its use in self-samples-based CC screening, particularly in low-resource settings that would benefit the most from this cancer prevention strategy. Badman and colleagues had investigated four non-volatile transport media as potential alternatives to ThinPrep ^®^PreservCyt for HPV screening by using HPV-infected cell lines ^5^. As eNat ^®^ is not flammable, is able to inactivate infectious agents present in the sample and stabilizes nucleic acids in samples stored at room temperature ^6, 17^. Moreover, it is already routinely used as a medium for molecular HPV detection ^8, 18^ and it could represents a valid alternative to ThinPrep ^®^PreservCyt ^®^. In addition, this study demonstrated an almost perfect agreement between vaginal swabs suspended in ThinPrep ^®^PreservCyt ^®^ and eNat ^®^ using the three different diagnostic assays. Data obtained using the quantitative HPV OncoPredict detection assay indicated that the discordances in HPV detection observed between the two vaginal swabs collected by each participating woman were related to the low viral load observed in the discordant samples (below 6.24E+02 cp/10,000 cells). Discrepant results had been previously reported in samples with low HPV viral load ^18^. Moreover, all the observed discordances were observed in patients with low-grade or negative cytology.

The mean hrHPV viral load for self-collected vaginal swabs, eluted in ThinPrep ^®^PreservCyt ^®^ and for those suspended in eNat ^®^, was higher (3.26E+05 cp/10,000 cells and 4.59E+05 cp/10,000 cells; respectively) than that observed in cervical samples (2.03E+05 cp/10,000 cells). This difference may be due to the higher number of exfoliating cells present in the vagina and/or to the differences in the suspension volumes of cervical and vaginal specimens (20 mL vs 5 mL), although viral load normalization based on the samples’ human cellularity was expected to have reduced differences associated with suspension volumes.

## Conclusion

In conclusion, this study demonstrated that vaginal self-sampling is a good alternative to cervical swab if the sample is collected in either ThinPrep ^®^PreservCyt ^®^ or eNat ^®^, with the second medium allowing viral inactivation and providing a good strategy to further reduce costs.

Previous studies compared different devices for vaginal self-sampling considering cost, simplicity of use and accuracy in HPV detection. FLOQSwab ^®^ appeared to be the best option because of its performances, cost and the possibility to transport samples dry ^18, 19^. To our knowledge, this is the first study that compares eNat ^®^ to ThinPrep ^®^PreservCyt ^®^ for the suspension of vaginal samples. Future studies including a greater number of clinical samples and other alternative suspension media are necessary to better evaluate the best solution for vaginal self-samples testing.

## Data availability

Zenodo: Human papillomavirus (HPV) detection in vaginal self-samples: evaluation of eNat ^®^ as an alternative suspension medium to ThinPrep ^®^PreservCyt ^®^ for vaginal swabs. https://doi.org/10.5281/zenodo.6077699 ^20^

This project contains the following files:

- HPV positivity on cervical and.csv
- hrHPV viral load.csv
- Legend HPV positivity on cervical and vaginal self-samples with different HPV real-time assay.docx
- Legend hrHPV viral load.docx

Data are available under the terms of the Creative Commons Attribution 4.0 International license (CC-BY 4.0).

## Author contributions

Conceptualization: Clementina Elvezia Cocuzza

Data curation: Chiara Giubbi, Marianna Martinelli, Clementina Elvezia Cocuzza, Ivan Vallini, Tarig Dafa'alla

Formal analysis: Chiara Giubbi

Funding acquisition: Clementina Elvezia Cocuzza, Paolo Romano, Winnie Wu

Investigation: Chiara Giubbi, Federica Perdoni, Tarig Dafa'alla, Silvia Paganoni

Methodology: Chiara Giubbi, Marianna Martinelli, Ivan Vallini, Silvia Paganoni, Santina Castriciano, Rosario Musumeci

Project administration: Clementina Elvezia Cocuzza

Resources: Paolo Romano, Winnie Wu, Clementina Cocuzza

Supervision: Clementina Elvezia Cocuzza

Visualization: Chiara Giubbi, Marianna Martinelli

Writing – original draft preparation: Chiara Giubbi, Marianna Martinelli

Writing – review & editing: Clementina Elvezia Cocuzza, Rosario Musumeci, Tarig Dafa'alla, Winnie Wu, Santina Castriciano, Paolo Romano.
