# Supplementary material for: Human papillomavirus (HPV) detection in vaginal self-samples: evaluation of eNat® as an alternative suspension medium to ThinPrep®PreservCyt® for vaginal swabs
Source: Open Res Eur. 2022 Jul 22;2:35. Originally published 2022 Mar 10. [Version 2] doi: 10.12688/openreseurope.14344.2 (PMC10446064; doi:10.12688/openreseurope.14344.2)
Supplement: Peer review history

# Peer review: Reviewer response for version 2

**DOI:** 10.21956/openreseurope.16153.r29753

**Published:** 2022-08-05

**Authors:** Alberto Severini

**Affiliations:** 1 Department of Medical Microbiology and Infectious Diseases, Faculty of Health Sciences, University of Manitoba, Winnipeg, Canada

The authors have addressed my minor comments satisfactorily and I have no more concerns.  I recommend that the manuscript be accepted for indexing without further revisions.

 Thank you for giving me the opportunity to review this manuscript.

Is the study design appropriate and does the work have academic merit?

Yes

Is the work clearly and accurately presented and does it cite the current literature?

Yes

If applicable, is the statistical analysis and its interpretation appropriate?

Partly

Are all the source data underlying the results available to ensure full reproducibility?

Yes

Are the conclusions drawn adequately supported by the results?

Yes

Are sufficient details of methods and analysis provided to allow replication by others?

Yes

Reviewer Expertise:

NA

I confirm that I have read this submission and believe that I have an appropriate level of expertise to confirm that it is of an acceptable scientific standard.

# Peer review: Reviewer response for version 2

**DOI:** 10.21956/openreseurope.16153.r29754

**Published:** 2022-08-01

**Authors:** David Hawkes

**Affiliations:** 1 VCS Foundation, Carlton South, Australia

Thank you to the authors for their responses. I am satisfied with these responses to my questions. The only other item I would raise is more for their own interest. The data for M0244 and M0222T12 showed that the PapilloCheck and Seegene assay produced hPV68 results in 5/6 and 6/6 specimens, however, the GeneFirst OncoPredict assay returned a HPV39 positive result for 5/6 specimens. Considering how genetically close the HPV68 and HPV39 HPV types are I might suggest a discussion with the manufacturers of that assay identifying a potential source of off-target responses (HPV39 positive result) in the presence of HPV68 (it would require more follow-up to determine whether this observation was for HPV68a and/or HPV68b).

Is the study design appropriate and does the work have academic merit?

Yes

Is the work clearly and accurately presented and does it cite the current literature?

Yes

If applicable, is the statistical analysis and its interpretation appropriate?

Yes

Are all the source data underlying the results available to ensure full reproducibility?

Yes

Are the conclusions drawn adequately supported by the results?

Yes

Are sufficient details of methods and analysis provided to allow replication by others?

Yes

Reviewer Expertise:

NA

I confirm that I have read this submission and believe that I have an appropriate level of expertise to confirm that it is of an acceptable scientific standard.

# Peer review: Reviewer response for version 1

**DOI:** 10.21956/openreseurope.15481.r28985

**Published:** 2022-04-12

**Authors:** Alberto Severini

**Affiliations:** 1 Department of Medical Microbiology and Infectious Diseases, Faculty of Health Sciences, University of Manitoba, Winnipeg, Canada

Giubbi *et al.* have compared the detection of HPV in self-collected samples transported in PreservCyt® and in Copan eNat® medium. The results show no difference in sensitivity between the two transport media.  These are useful results, which demonstrate the utility of the aqueous eNat® medium for self-collected HPV specimens.

 Minor comments:

1. The weakness of this study is the low number of women (n=30). The study does not have the power to detect small differences in performance that may matter when hundreds of thousands of women are tested. The authors should point this out in their discussion.
2. The authors compare the HPV viral loads detected in various types of specimens and conclude that vaginal specimens have a lower viral load. However, there is no statistical analysis to support this conclusion and it is possible that the small differences observed fall within the expected experimental variance.  

Is the study design appropriate and does the work have academic merit?

Yes

Is the work clearly and accurately presented and does it cite the current literature?

Yes

If applicable, is the statistical analysis and its interpretation appropriate?

Partly

Are all the source data underlying the results available to ensure full reproducibility?

Yes

Are the conclusions drawn adequately supported by the results?

Yes

Are sufficient details of methods and analysis provided to allow replication by others?

Yes

Reviewer Expertise:

Molecular diagnostics, HPV molecular epidemiology, Vaccine preventable diseases

I confirm that I have read this submission and believe that I have an appropriate level of expertise to confirm that it is of an acceptable scientific standard, however I have significant reservations, as outlined above.

# Response

**Published:** 2022-06-30

**Authors:** Clementina COCUZZA

The authors would like to thank Prof. Alberto Severini for his review and comments. (Reviewer comments in italics)

*Giubbi et al. have compared the detection of HPV in self-collected samples transported in PreservCyt® and in Copan eNat® medium. The results show no difference in sensitivity between the two transport media.  These are useful results, which demonstrate the utility of the aqueous eNat® medium for self-collected HPV specimens.*

*Minor comments:*

- *The weakness of this study is the low number of women (n=30). The study does not have the power to detect small differences in performance that may matter when hundreds of thousands of women are tested. The authors should point this out in their discussion.*

**Reply:** We agree with the reviewer that the main limitation of this study is the number of women enrolled. As reported in the text of the manuscript, this is intended to be a pilot study aiming to perform a preliminary evaluation of a possible alternative suspension medium for vaginal-self samples. This eNAT medium by Copan has as intended use the resuspension of clinical samples for other molecular tests, such as microbiome analysis, and studies on its use for this purpose have also been previously published. However future studies on a larger number of women will be necessary to better evaluate the performance of eNAT in vaginal sample resuspension for HPV testing in order to evaluate if any difference in high risk HPV detection is related to the suspension medium.  

- *The authors compare the HPV viral loads detected in various types of specimens and conclude that vaginal specimens have a lower viral load. However, there is no statistical analysis to support this conclusion and it is possible that the small differences observed fall within the expected experimental variance.*

**Reply**: Due to the restricted number of samples analysed no statistical analysis was performed. However, vaginal self-samples suspended in both suspension media demonstrated to have a higher viral load than cervical specimens. As reported in the text, this finding may be partially related to the different suspension volume (5 ml vs 20 ml) used for the two different sample types, but this finding needs to be confirmed by a larger study which would also allow to determine whether the difference in viral load between cervical and vaginal samples is statistically significant.

# Peer review: Reviewer response for version 1

**DOI:** 10.21956/openreseurope.15481.r28790

**Published:** 2022-03-24

**Authors:** David Hawkes

**Affiliations:** 1 VCS Foundation, Carlton South, Australia

The study but Giubbi *et al* is interesting and makes a good step forward for the development of a consistent protocol for self-collection for the purpose of PV-based cervical screening. There are a few minor comments for consideration:

- There may be differences in the samples stored at -20C depending on whether they were in Hologic PreservCyt or Copan eNAT. PreservCyt is alcohol based and generally does not freeze at -20C whereas it would be likely that eNAT would freeze. A freeze thaw cycle is likely to only have been experienced by the samples in eNat which may affect the sample, a freeze thaw cycle may break down DNA or alternatively it may lyse cellular material freeing more DNA . The authors need to address this issue.
- The authors should include a table showing the HPV results per sample (e.g. simply whether the same was positive for HPV or negative for each assay). These data are referred to in the results section and in Table 1. These data would show whether there were any trends in samples (e.g. if the 15 GeneFirst positive samples were also positive on the Seegene and OncoPredict assays - or whether the differences observed were associated with particular HPV types). This would also identify if there were any trends with comparison between the three specimen types.
- The authors need to address that there appears to be a trend (unclear whether it is significant) for lower sensitivity across all sample types for the GeneFirst assay. The Seegene assay has been clinically validated and could be utilised as a comparator assay for the purpose of determining sensitivity and specificity for the two emerging assays. 

Is the study design appropriate and does the work have academic merit?

Yes

Is the work clearly and accurately presented and does it cite the current literature?

Yes

If applicable, is the statistical analysis and its interpretation appropriate?

Yes

Are all the source data underlying the results available to ensure full reproducibility?

Yes

Are the conclusions drawn adequately supported by the results?

Yes

Are sufficient details of methods and analysis provided to allow replication by others?

Yes

Reviewer Expertise:

HPV diagnostics

I confirm that I have read this submission and believe that I have an appropriate level of expertise to confirm that it is of an acceptable scientific standard, however I have significant reservations, as outlined above.

# Response

**Published:** 2022-04-06

**Authors:** Clementina COCUZZA

The authors would like to thank Prof. David Hawkes for his review and comments. Below we have included the response to his comments for consideration:

- *There may be differences in the samples stored at -20C depending on whether they were in Hologic PreservCyt or Copan eNAT. PreservCyt is alcohol based and generally does not freeze at -20C whereas it would be likely that eNAT would freeze. A freeze thaw cycle is likely to only have been experienced by the samples in eNat which may affect the sample, a freeze thaw cycle may break down DNA or alternatively it may lyse cellular material freeing more DNA. The authors need to address this issue.*

**Reply: **We thank Prof. David Hawkes for this consideration related to the preanalytical process. As indicated in the “Method” section, in the paragraph on “Pre-analytical sample processing”, it was stated that processing of cervical samples was performed as follows: “In the laboratory all specimens were vortexed for 30 seconds, and subsequently 1.5 ml aliquots were dispensed in cryovials; one was used for DNA extraction and the others stored at -20°C.” Similarly, vaginal samples were processed as follows: “Five aliquots of 1 ml were made from each of the vaginal specimens; one was used for nucleic acids extraction and the others stored at -20°C.” All samples were therefore analyzed fresh and nucleic acid extraction performed on a sample aliquot prior to freezing and therefore results were not affected by a freeze thaw cycle.

- *The authors should include a table showing the HPV results per sample (e.g. simply whether the same was positive for HPV or negative for each assay). These data are referred to in the results section and in Table 1. These data would show whether there were any trends in samples (e.g. if the 15 GeneFirst positive samples were also positive on the Seegene and OncoPredict assays - or whether the differences observed were associated with particular HPV types). This would also identify if there were any trends with comparison between the three specimen types.*

**Reply:** File csv reporting all results with all investigated assays for each study sample is available at the link: https://doi.org/10.5281/zenodo.6077699. In particular, you will find the results of the 3 different assays for all samples in the file entitled “HPV positivity on cervical and vaginal samples.cvs and relative file including “Legend of HPV positivity on cervical and vaginal samples.docx”. The Open Research Europe journal requires all the data to be open access but limits the number of tables/figures. *However in reply to *Prof. David Hawkes request we include an additional summary table reporting all the HPV results obtained from the analyzed samples. This table can be seen here.

- *The authors need to address that there appears to be a trend (unclear whether it is significant) for lower sensitivity across all sample types for the GeneFirst assay. The Seegene assay has been clinically validated and could be utilized as a comparator assay for the purpose of determining sensitivity and specificity for the two emerging assays.*

**Reply:** We agree with Prof. David Hawkes comment regarding the lower HPV positivity reported from GeneFirst test compared to the other two. We have reported these results in the manuscript. As you can see in the above table the discordances of HPV positivity in cervical sample are present in two samples (MO180 T18 and MO222 T12). Both samples were collected from women with a cytology result of low-grade cervical dysplasia who did not require a biopsy to be performed at colposcopy based on the clinical findings. Regarding vaginal self-samples, the discrepancy in the HPV detection were shown in only one sample (MO149 T24). In this case the associated cytology was negative for the presence of any cervical alterations.
